# Endoscopic management of gastric perforation secondary to chicken bone: A report of 2 cases

**DOI:** 10.1016/j.ijscr.2019.11.010

**Published:** 2019-11-15

**Authors:** M Masood Sidiqi, Siddhanth Sharma, Ausama H Muhammed

**Affiliations:** General Surgery, Bunbury Regional Hospital, Western Australia, Australia

**Keywords:** Foreign-body, Gastric, Perforation, Endoscopy, Complication

## Abstract

**Introduction:**

Ingested foreign bodies (IFB) can uncommonly cause perforation of the gastrointestinal tract. The traditional management is surgical exploration via laparotomy or laparoscopy, although endoscopic options are now gaining prominence.

**Presentation of case:**

We present two patients with almost identical clinical presentations of post-prandial abdominal pain and anorexia. On examination they were haemodynamically stable with localised epigastric tenderness. Both patients underwent CT scan of the abdomen, with one scan revealing a foreign body in the stomach penetrating the full thickness of the gastric wall with the tip lying extraluminally. They subsequently underwent endoscopy where a chicken bone was found perforating the wall of the stomach. This was removed via snare and endoscopic clips were used to close the site of perforation.

**Discussion:**

The majority of ingested foreign bodies pass through the gastrointestinal tract harmlessly. However some IFBs can cause significant complications like bowel obstruction, bleeding, abscess formation, migration to other organs, and in our case perforation. The diagnosis may be delayed due to an insidious clinical presentation especially if the patient does not recall ingesting anything untoward. Our patients managed to avoid surgery by undergoing successful endoscopic therapy.

**Conclusion:**

In selected cases, endoscopic management is more cost-effective, minimally invasive, has less post-operative complications, and leads to a more expeditious recovery. Therefore, the role of therapeutic endoscopy for gastric perforations secondary to foreign bodies should always be considered.

## Highlights

• The majority of ingested foreign bodies pass through the gastrointestinal tract harmlessly.
• Ingested foreign bodies can cause significant complications like bowel obstruction, bleeding, abscess formation, migration to other organs, and perforation.
• In selected cases, endoscopic management is more cost-effective, minimally invasive, has less post-operative complications, and leads to a more expeditious recovery.

## 1 Introduction

The gastrointestinal tract is a relatively resilient apparatus with its ability to facilitate the safe passage of ingested foreign bodies (IFBs). The majority of IFBs pass uneventfully without causing any harm and are excreted in the stool within 1 week, while about 1 % will cause perforation [1,2]. This includes dietary foreign bodies like fish bones, chicken bones, and shell fragments. They may induce various clinical manifestations such as bowel obstruction, bleeding, abscess formation, and migration to other organs [3]. Immediate surgical intervention (laparoscopic vs open) is the traditional treatment of choice for frank gastrointestinal perforation. However the role of endoscopic management is certainly gaining recognition [4]. To the best of our knowledge, there have only been 4 published case reports of successful endoscopic removal of perforating gastric foreign bodies [[5], [6], [7], [8]]. We report on two patients that had penetrating chicken bones removed endoscopically, with clips used to close the penetration site. This work has been reported in line with the SCARE criteria [9].

## 2 Presentation of case

### 2.1 Case 1

A 58 year old gentleman presented to the emergency department with a two month history of worsening epigastric pain. He explained the pain was worse after meals, sharp and throbbing in nature, lasting between fifteen and thirty minutes. He had recently started on Ibuprofen for back pain. Other than being obese, he had no significant past medical history. On examination he was hemodynamically stable. His abdomen was soft and tender in the epigastric/ left upper quadrant.

**Fig. 1:**
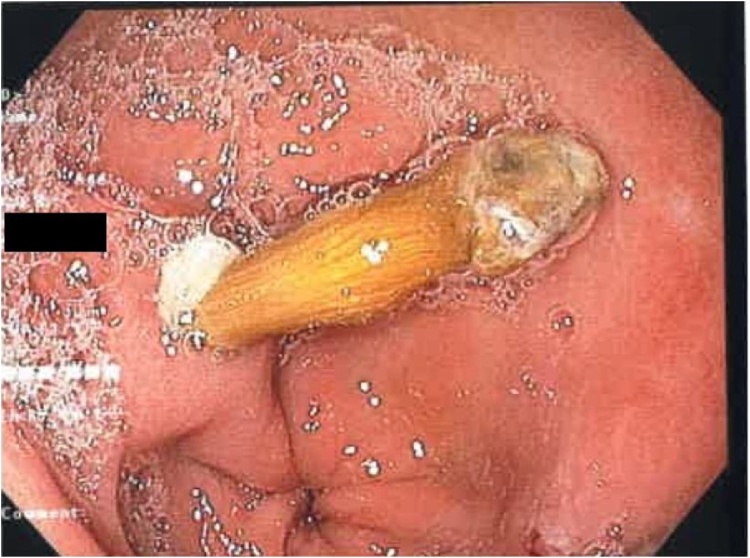
Chicken bone seen perforating gastric wall.

**Fig. 2:**
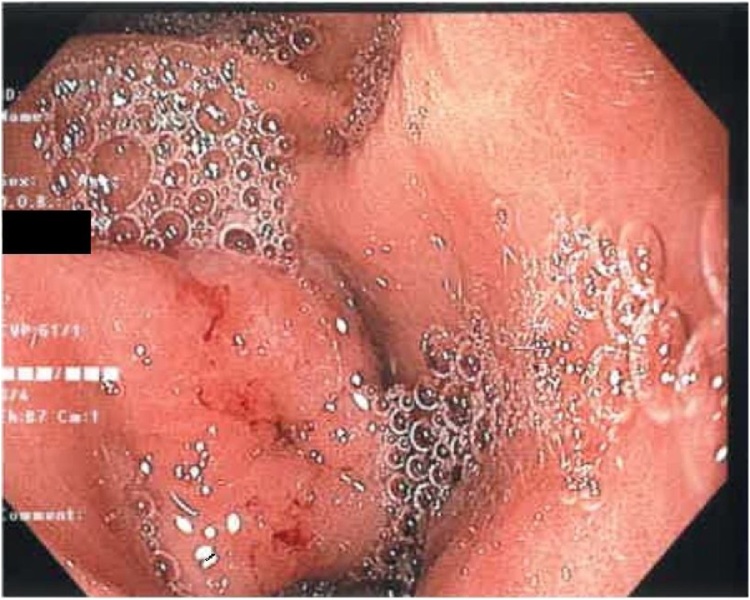
Site of perforation after chicken bone removed.

**Fig. 3:**
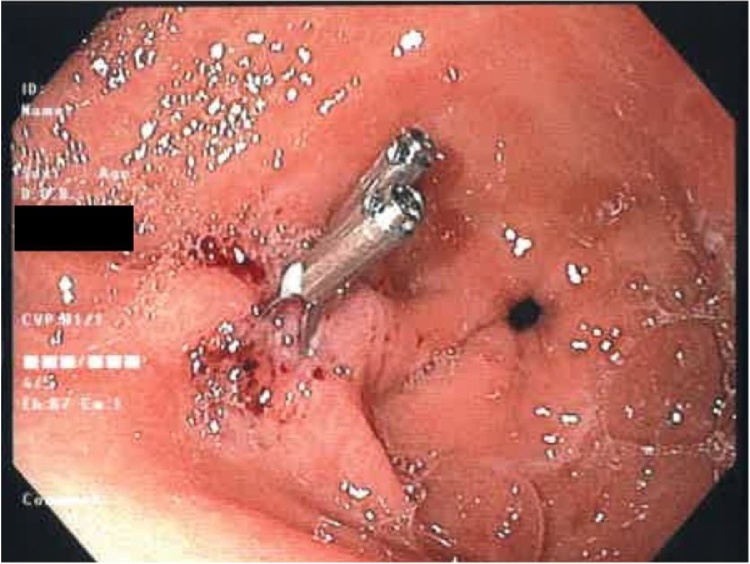
Site of perforation closed with clips. Pylorus also seen on the right of the image.

**Fig. 4:**
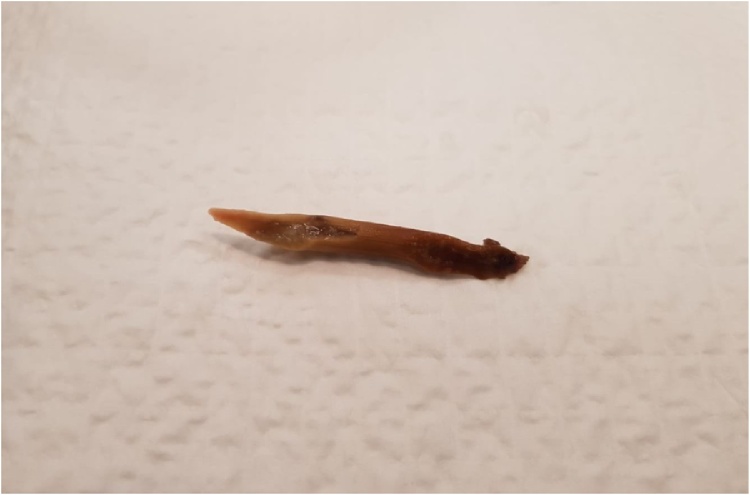
The culprit.

The patient’s inflammatory markers were elevated with a white cell count of 17.4 × 10^9^/L (normal 4−11 × 10^9^/L) and a C reactive protein of 11 mg/L (normal <5 mg/L). His other blood tests including haemoglobin, creatinine, liver function, lipase and troponin were within normal limits. Computed tomography scan of the abdomen showed minor non-specific fat stranding at the distal stomach and proximal duodenum suggesting a duodenitis, which fit in with the recent history of non-steroidal anti-inflammatory medication use. The patient was admitted to hospital and started on a proton pump inhibitor. During his admission he spiked multiple fevers and became tachycardic. Multiple investigations were performed to look for the source of sepsis, including ECHO and ultrasound of the gallbladder, but to no avail. A repeat CT abdomen was ordered 48 h later as the patient failed to improve clinically. On this occasion, although no obvious source of sepsis was found, a thin feint opacity was noticed in the distal aspect of the stomach. Subsequently, the patient underwent an oesophagogastroduodenoscopy. A 4 cm sharp animal bone was found to be penetrating the inflamed antral mucosa. It was successfully removed with a snare and the site of perforation was closed with 3 endoscopic haemoclips clips Fig. 1, Fig. 2, Fig. 3, Fig. 4.

The patient improved significantly after the procedure, experiencing no further pain or fevers. He was discharged on day 7 with a short course of amoxicillin/clavulanic acid and pantoprazole 40 mg BD for 3 months.

### 2.2 Case 2

**Fig. 5:**
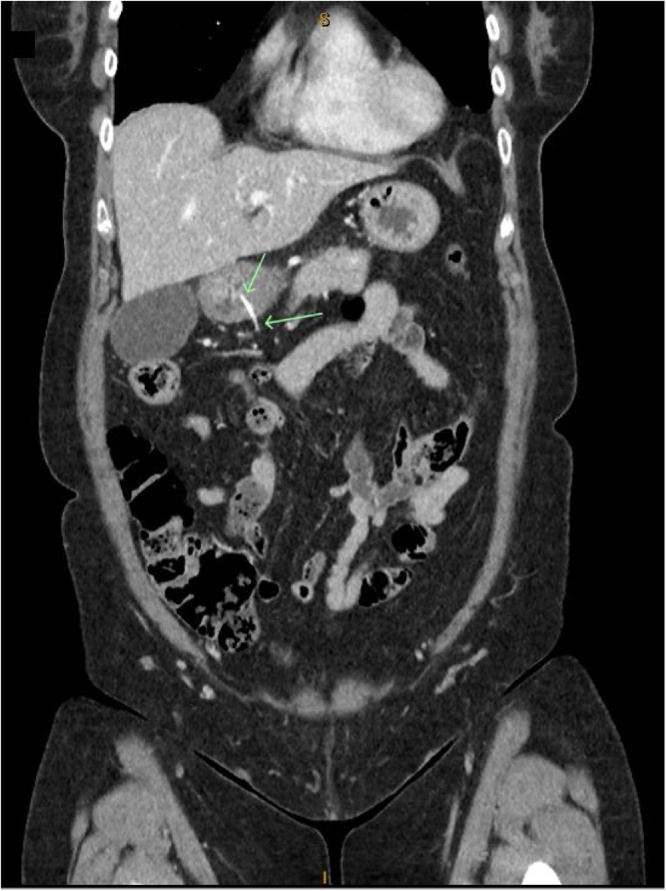
CT abdomen revealed a calcified foreign body perforating the gastric wall.

An 80 year old lady presents with an almost identical case as the patient presented above. She complained of abdominal pain that was worse after meals. The onset of pain was two nights prior after having dinner which contained chicken bones. On examination she was haemodynamically stable with a soft abdomen, but had a significantly tender epigastrium. Computed tomography scan of the abdomen showed a 4 cm foreign body that was perforating the full thickness of the gastric wall with its tip lying outside the lumen. There was no evidence of extra-luminal air, although some free fluid was noted around the stomach Fig. 5.

After being started on a proton pump inhibitor and antibiotics, the patient was taken to the operating room for oesophagogastroduodenoscopy. In similar fashion to the above case, the chicken bone was snared and removed and 2 endoscopic clips were used to close the site of perforation. She experienced immediate relief of her symptoms and after 48 h of observation was discharged home on oral pantoprazole.

## 3 Discussion

Although IFBs are common in children, they are infrequently seen in adult prison inmates, psychiatric patients, alcoholics, elderly individuals with dentures, and selected professions, like carpenters and dressmakers, who tend to hold small sharp objects in their mouths [10]. Dietary food particles such as fish bones, bone fragments and vegetable-fibre bezoars are the most frequently ingested foreign bodies [11]. Objects that are thin, long and sharp are more likely to cause damage including needles, toothpicks and fish bones [2]. Ingested batteries are also concerning due to their potential to induce exothermal burns and pressure necrosis. IFB’s can lodge at any site of the gastrointestinal tract, but are more frequently seen at areas of physiological narrowing including the oesophageal sphincters, pylorus, ileocaecal valve, sigmoid colon and anus. Furthermore, areas of acute angulation are more likely to perforate, such as the ileocaecal valve and rectosigmoid regions [10,11].

Foreign body related perforations occurring in the stomach, duodenum, and large intestine present insidiously as seen in the presented cases, often leading to delayed presentation, diagnosis, and subsequent management. This is thought to be due to the characteristic thick muscular walls of these sections of the gastrointestinal tract and the presence of surrounding omentum. Accordingly, a gradual perforation usually occurs while the concurrent sealing effect of the surrounding tissues will eventually lead to intra-abdominal abscesses in many cases [7]. Furthermore, the diagnosis is made harder with the patient’s inability to recall the consumption of a foreign body.

Given the majority of IFBs pass through naturally, conservative treatment is justified in most cases. Intervention is typically indicated when the object is deemed to be long and sharp, or there is clinical or radiological evidence that complications have developed [12]. Traditionally, frank gastrointestinal perforation required immediate surgical intervention either via laparoscopy or laparotomy. However since Binmoeller successfully closed an iatrogenic gastric perforation via endoscopy using haemoclips in 1993, a device originally created in Japan to control upper GI haemorrhage, the paradigm is slowly changing [13]. Endoscopic interventions are attractive due to their reduced cost and minimally invasive nature, minimising the risk of post-operative complications and allowing more expeditious recovery [12]. Contraindications to endoscopic therapy include peritonitis, obstruction, bleeding or severe inflammation in the abdominal cavity, penetration to vessels, and migration to other organs. In the English literature there have only been 4 cases published of successful endoscopic management of gastric perforation secondary to ingested foreign bodies [[5], [6], [7], [8]]. Boškoski and colleagues closed a duodenal perforation caused by a 12 cm spoon endoscopically, utilising 5 haemoclips and subsequently injecting 3 ml of fibrin glue to consolidate the closure [14]. Likewise more recently, a lollipop stick that had perforated the duodenum, ingested two weeks before presentation, was removed using endoscopic forceps and closed via haemoclips and a detachable snare [15]. Our current limited literature and preliminary results suggest endoscopic techniques can be useful when the diameter of the perforation is less than the width of the clip’s nail, the edges are smooth, and the perforation is clearly visible [[13], [14], [15], [16]]. Further studies are required to develop recommendations and definitive guidelines.

## 4 Conclusion

In selected cases, the role of therapeutic endoscopy for gastric perforations secondary to foreign bodies should be considered.

## Funding

None.

## Ethical approval

Ethical approval is not applicable.

## Consent

Written informed consent was obtained from the patient for publication of this case report and accompanying images. A copy of the written consent is available for review by the Editor-in-Chief of this journal on request.

## Author contribution

Dr Masood Sidiqi and Dr Siddhanth Sharma contributed in medical record review, literature search, and writing of the draft. Dr Ausama Muhammed contributed towards review of the paper.

## Registration of research studies

NA.

## Guarantor

All authors have read and approved the manuscript and accept full responsibility for the work.

## Provenance and peer review

Commissioned, externally peer-reviewed

## Declaration of Competing Interest

Authors have no conflict of interest to disclose.
